# Loss of CLCA4 Promotes Epithelial-to-Mesenchymal Transition in Breast Cancer Cells

**DOI:** 10.1371/journal.pone.0083943

**Published:** 2013-12-26

**Authors:** Yang Yu, Vijay Walia, Randolph C. Elble

**Affiliations:** 1 Department of Pharmacology, Southern Illinois University School of Medicine, Springfield, Illinois, United States of America; 2 Simmons Cancer Institute, Southern Illinois University School of Medicine, Springfield, Illinois, United States of America; 3 Laboratory of Cell and Developmental Signaling, Center for Cancer Research, National Cancer Institute-Frederick, Frederick, Maryland, United States of America; University of North Carolina School of Medicine, United States of America

## Abstract

The epithelial to mesenchymal transition (EMT) is a developmental program in which epithelial cells downregulate their cell-cell junctions, acquire spindle cell morphology and exhibit cellular motility. In breast cancer, EMT facilitates invasion of surrounding tissues and correlates closely with cancer metastasis and relapse. We found previously that the candidate tumor suppressor CLCA2 is expressed in differentiated, growth-arrested mammary epithelial cells but is downregulated during tumor progression and EMT. We further demonstrated that CLCA2 is a p53-inducible proliferation-inhibitor whose loss indicates an increased risk of metastasis. We show here that another member of the CLCA gene family, CLCA4, is expressed in mammary epithelial cells and is similarly downregulated in breast tumors and in breast cancer cell lines. Like CLCA2, the gene is stress-inducible, and ectopic expression inhibits colony formation. Transcriptional profiling studies revealed that CLCA4 and CLCA2 together are markers for mammary epithelial differentiation, and both are downregulated by TGF beta. Moreover, knockdown of CLCA4 in immortalized cells by shRNAs caused downregulation of epithelial marker E-cadherin and CLCA2, while mesenchymal markers N-cadherin, vimentin, and fibronectin were upregulated. Double knockdown of CLCA2 and CLCA4 enhanced the mesenchymal profile. These findings suggest that CLCA4 and CLCA2 play complementary but distinct roles in epithelial differentiation. Clinically, low expression of CLCA4 signaled lower relapse-free survival in basal and luminal B breast cancers.

## Introduction

Metastatic breast cancer remains a largely intractable disease. Most relapses are attributable to the basal subtype, which is typified by the loss of epithelial markers [1]–[4]. The reversal of epithelial differentiation to a mesenchymal, stem cell-like state is considered one of the hallmarks of tumor progression [5]. Indeed, epithelial to mesenchymal transition, EMT, affords several advantages to the evolving tumor, conferring invasiveness, growth-factor independence, and resistance to many forms of stress including chemotherapy [4], [6]–[8]. Understanding and potentially inhibiting this process is a fundamental goal of breast cancer research [9]–[11].

Homeostasis of epithelial tissues is maintained by signaling pathways that depend on structural features of the tissue itself. For example, loss of E-cadherin from cell-cell junctions unleashes a cascade of events leading to EMT [8]. Dysregulation of ion currents can also promote EMT. For example, upregulation of the chloride/potassium co-transporter KCC-3 is associated with invasiveness in cervical cancer, and its ectopic expression drives EMT [12].

The human genome encodes three functional chloride channel accessory (CLCA) proteins, but only two are expressed in mammary epithelium, CLCA2 and CLCA4 [13]–[15]. We showed previously that CLCA2 is a p53-inducible inhibitor of cell proliferation and that it is a marker of differentiated epithelium that is downregulated with tumor progression [15], [16]. Ectopic expression of CLCA2 inhibited proliferation while knockdown caused EMT [15], [16].

CLCA4 is predominantly expressed in colon, along with another member of the CLCA family, CLCA1 [14]. Both are precipitously downregulated with tumor progression (it should be noted that CLCA4 was misidentified as CLCA2 in that study [17]). While CLCA1 has been shown to be a proliferation inhibitor in colon cell lines, the role of CLCA4 remains unexplored in colon or breast [18].

In this study, we sought to determine whether CLCA4, like CLCA2, contributes to differentiation in breast. We found that CLCA4 was similarly downregulated in breast cancer, that its ectopic expression inhibited breast cancer cell proliferation, and that CLCA4 knockdown induced EMT in mammary epithelial cells. These results suggest that different CLCA family members may perform distinct functions in the same cell to maintain epithelial differentiation.

## Results

### CLCA4 is a proliferation-inhibitor that is frequently downregulated in human cancers

**Figure 1:**
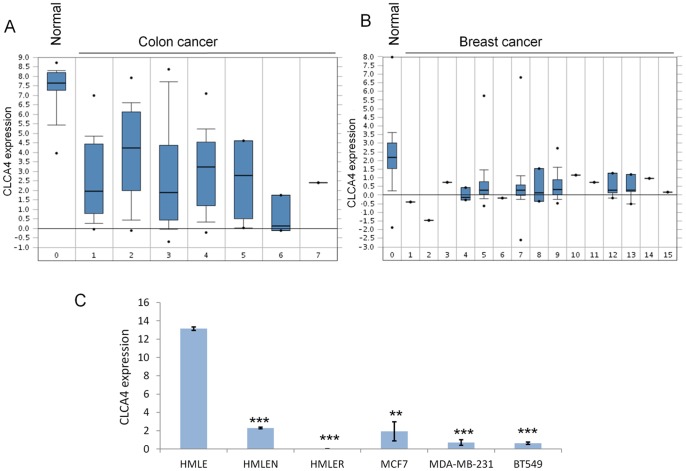
CLCA4 downregulation in colon and breast cancers. **A** and **B**, CLCA4 mRNA expression in normal tissue compared to cancer in colon/rectum and breast. The Cancer Genome Atlas (TCGA) datasets were searched using Oncomine. The log2 median-centered ratios for CLCA4 expression level are depicted in box-and-whisker plots. Dots represent maximum and minimum outliers from the main dataset. For each plot, the following pathological subtypes were evaluated separately. **A**, colorectal: 0, normal tissue (22); 1, cecum adenocarcinoma (22); 2, colon adenocarcinoma (101); 3, colon mucinous adenocarcinoma (22); 4, rectal adenocarcinoma (60); 5, rectal mucinous adenocarcinoma (6); 6, rectosigmoid adenocarcinoma (3); 7, rectosigmoid mucinous adenocarcinoma (1). **B**, breast: 0, normal tissue (61); 1, apocrine carcinoma (1); 2, large cell neuroendocrine (1); 3, ductal carcinoma (1); 4, intraductal cribriform adenocarcinoma (3); 5, invasive carcinoma (76); 6, invasion cribriform carcinoma (1); 7, invasive ductal carcinoma (395); 8, invasive ductal and lobular carcinoma (3); 9, invasive lobular breast carcinoma (36); 10, invasive papillary breast carcinoma (1); 11, metaplastic breast carcinoma (1); 12, mixed lobular and ductal breast carcinoma (7); 13, mucinous breast carcinoma (4); 14, papillary carcinoma (1); 15, pleomorphic carcinoma (1). Number in parentheses indicates sample size for each category. **C**, CLCA4 mRNA downregulation in breast cancer cell lines. RT-qPCR data from cultured cells.

To confirm previous observations and determine whether CLCA4 was downregulated in breast cancer as reported for colon cancer, we compared CLCA4 expression patterns in a curated database, The Cancer Genome Atlas (TCGA), using Oncomine. In accordance with Bustin [17], CLCA4 was downregulated in all colon tumor samples relative to normal (Figure 1A). TCGA revealed a similar loss of expression for breast cancer across all subtypes (Figure 1B). To further examine the pattern of loss, we performed RT-qPCR on well characterized breast cell lines. MDA-MB-231 and BT549 showed more than 99% downregulation relative to immortalized mammary epithelial cells, HMLE (Figure 1C). Transforming HMLE with oncogenes Her2 (HMLEN) or Ras (HMLER) caused precipitous downregulation of CLCA4 (Figure 1C, left).

**Figure 2:**
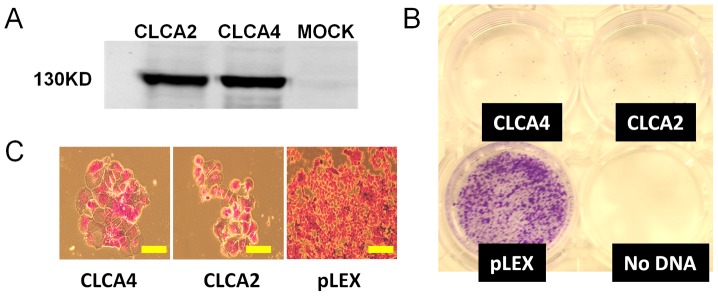
CLCA4 expression inhibits breast cancer cell proliferation. **A** western blot showing expression of Flag-tagged CLCA4 and CLCA2 transfected into 293 T cells. **B**, clonogenicity assays. CLCA4, CLCA2, and pLex vector were packaged and transduced into MCF7, and colonies were selected with puromycin for two weeks then stained with crystal violet in methanol. **C**, microimages of the surviving colonies. Bar, 200 microns. Data in B and C are representative of three repeats. The well marked “No DNA” was a non-transduced control for puromycin selection.

The frequent loss of CLCA4 expression with tumor progression suggested that CLCA4, like CLCA2, might antagonize tumorigenesis. We tested whether ectopic expression of CLCA4 inhibited tumor cell proliferation by lentivirally transducing Flag-tagged CLCA4 (Figure 2A) into MCF7 breast cancer cells and assessing the ability of the cells to form colonies. We found that both colony size and number were dramatically reduced by CLCA4 in parallel with CLCA2 (Figure 2B). Another breast cancer cell line, CA1d, and colon cancer cell line HCT116 responded similarly (Figure S1). MCF7 cells transduced with CLCA4 or CLCA2 produced microcolonies of enlarged cells (Figure 2C).

### CLCA4 is induced by cell stress

**Figure 3:**
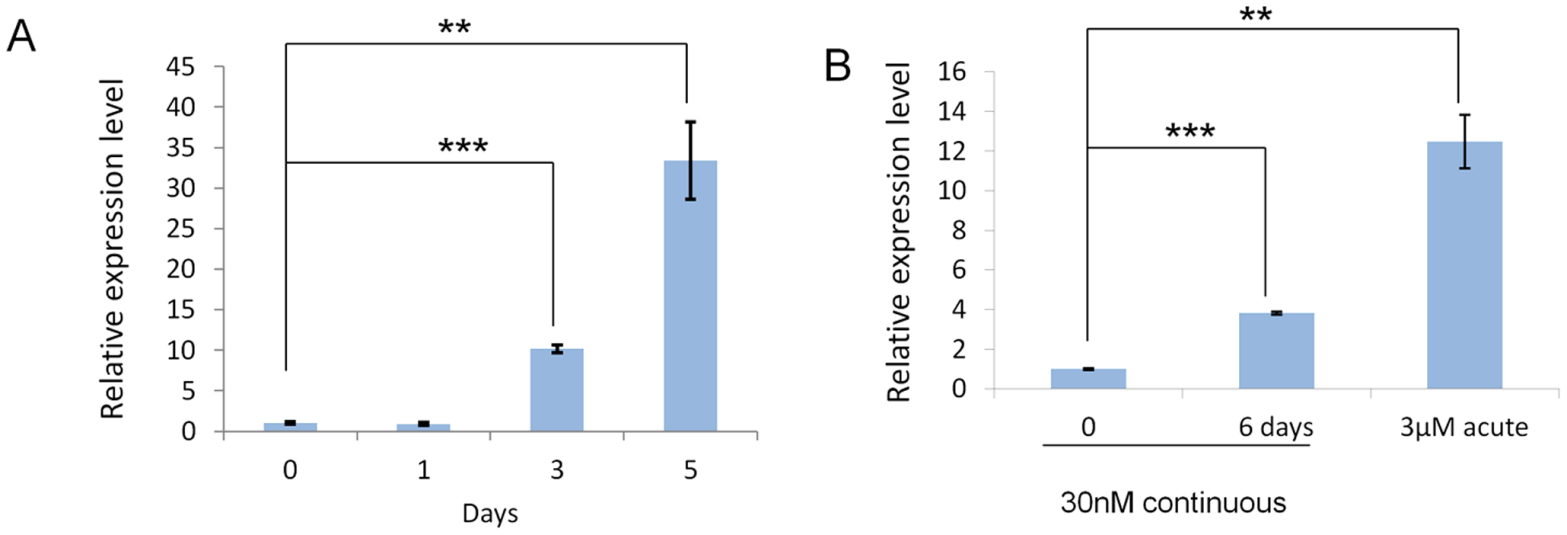
CLCA4 is moderately induced by doxorubicin. **A**, induction of CLCA4 mRNA in HMLE treated with 30 nM doxorubicin measured by RT-qPCR. **B**, induction of CLCA4 in MCF7. Cells were treated with 30 nM doxorubicin continuously for 6 days or 3 µM for 2 h then released into fresh medium for 2 days.

We found previously that CLCA2 transcription was induced by DNA damaging agents and other stressors [15]. To determine whether the same was true of CLCA4, we treated HMLE and MCF7 with doxorubicin. In HMLE, CLCA4 was induced up to 33-fold by treatment with 30 nM doxorubicin (Figure 3A). In comparison, CLCA2 was induced by 600-fold in the same experiment (data not shown). In MCF7, expression was more strongly induced by a short-term exposure to a high dose, 3 µM (Figure 3B). This acute exposure more closely mimics the administration of chemotherapy to cancer patients [19]. These data indicate that CLCA4 is induced by stress but more weakly than CLCA2.

### CLCA4 is a differentiation marker for breast epithelial cells

To determine whether CLCA4 is like CLCA2 a marker of epithelial differentiation in breast, we used Oncomine and NextBio to consult transcriptional profiles from cell culture systems that model mammary epithelial differentiation. In the first study, primary human mammary epithelial cells were cultured in three-dimensional Matrigel to promote epithelial differentiation or grown as mammospheres to promote stem-like properties [20]. Expression of both CLCA4 and CLCA2 was appoximately eightfold higher in the Matrigel population (Table 1). In the second study, the immortalized mammary epithelial cell line MCF10A was cultured in monolayer on plastic or on permeable membranes that support normal apico-basal polarization, barrier formation, and other aspects of differentiation [21]. CLCA4 expression was 56-fold higher and CLCA2 was seven times higher in the differentiated population [21].

**Table 1:** Association with epithelial differentiation.

	mRNA Fold Enrichment	
	CLCA4	CLCA2
Matrigel vs. mammospheres^1^	7.92+/−3.37	7.74+/−3.98
Barrier establishment^2^	56.8	7

^1^ Average of 2 experiments +/− standard deviation.

^2^ P = 0.0023.

To confirm these results in our own laboratory, we took advantage of an *in vitro* model for investigating mammary cell differentiation using HMLE. These cells are known to form cobblestone-like islands that exhibit an epithelial phenotype surrounded by cells that are mesenchymal in expression profile and behavior [6], [22]. We demonstrated recently that the islands are very resistant to trypsinization while mesenchymal cells detach readily. This property allows subpopulations to be separated by differential adhesion to substrate and profiled [22].

**Figure 4:**
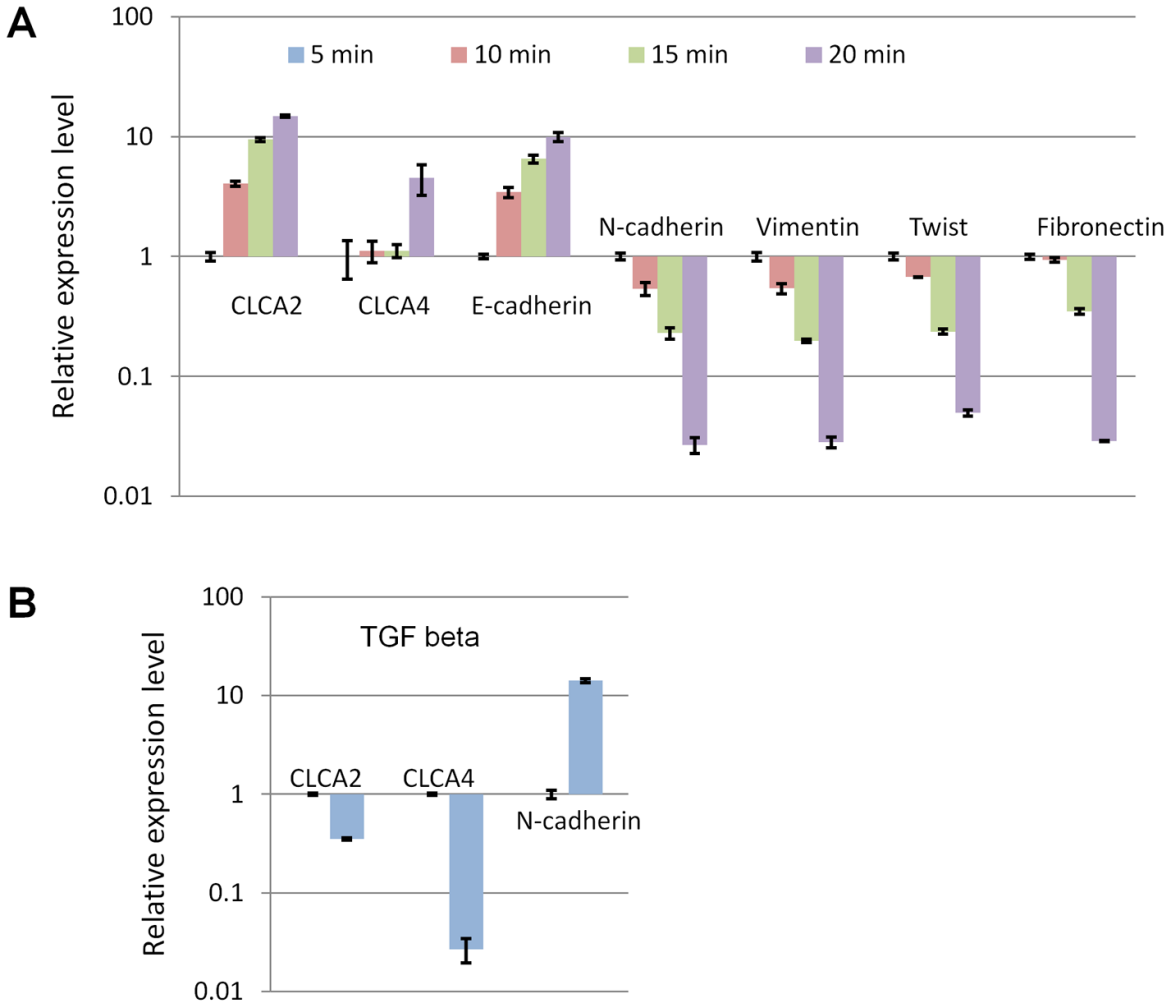
Expression of CLCA4 correlates with epithelial differentiation. **A**, HMLE cells were separated into epithelioid and mesenchymal subpopulations by differential trypsinization and subjected to RT-qPCR. The transcriptional profile reveals that CLCA4 is highest in the most trypsin-resistant fraction, correlating with E-cadherin and CLCA2. The 5 min fraction was normalized as 1. P<0.001 for all comparisons between 5 min and 20 min samples. **B**, EMT induced by TGF-beta treatment (2.5 ng/ml) downregulates CLCA4 and CLCA2. Values are normalized to no-drug control. P<0.01 for each comparison to no-drug control.

Accordingly, the most trypsin-resistant fraction had the highest expression of epithelial markers E-cadherin and CLCA2 and the lowest expression of mesenchymal markers such as vimentin and N-cadherin (Figure 4A). This fraction also had the highest expression of CLCA4. Together, these results indicate that CLCA4, like E-cadherin and CLCA2, is a marker of epithelial differentiation that is lost when cells transition to a mesenchymal phenotype. To test whether CLCA4 is repressed when cells undergo EMT, we treated HMLE with an agent that is known to induce EMT in these cells, TGF beta. Expression of CLCA4 declined by more than 90% while expression of the mesenchymal marker N-cadherin increased by the same factor (Figure 4B).

### Knockdown of CLCA4 promotes cell migration and invasion by inducing EMT

**Figure 5:**
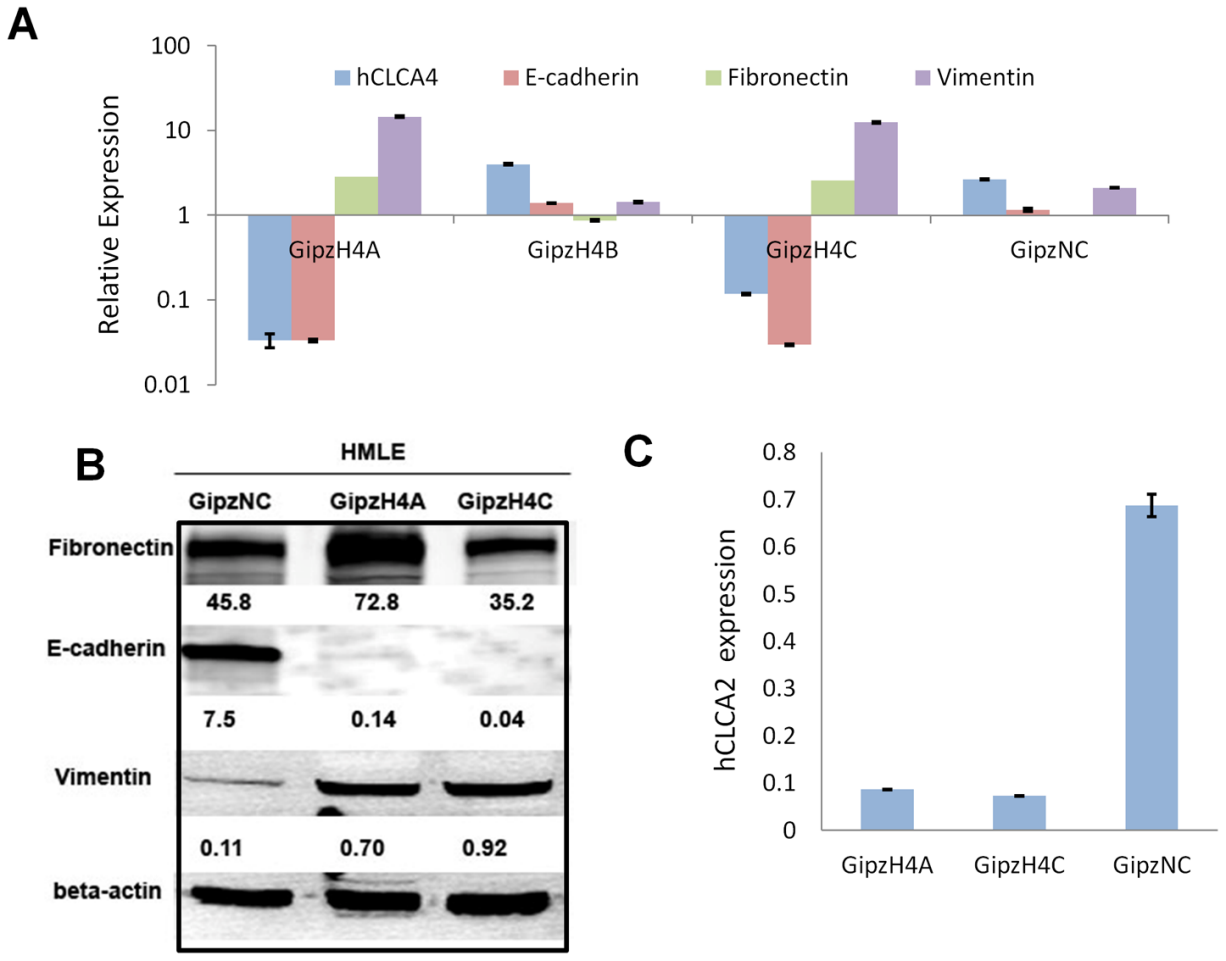
Knockdown of CLCA4 induces EMT. HMLE cells were lentivirally transduced with shRNAs GipzH4A, GipzH4B and GipzH4C. GipzNC contained a non-silencing control. **A**, transcriptional profile of CLCA4 and EMT markers. Values were normalized to HMLE parent. P<0.01 for each pairwise comparison between GipzNC and GipzH4A or GipzH4C. **B**, immunoblot of whole cell lysates probed for EMT marker proteins. Band intensities in each lane were normalized to the beta actin value. **C**, CLCA2 downregulation in CLCA4-knockdown cells. Values normalized to HMLE parent. P<0.001.

To determine whether CLCA4 was required for mammary epithelial cell differentiation, we used lentiviral shRNA knockdown to attenuate its expression in HMLE (GIPZ, OpenBiosystems). Three inserts were transduced and cell lines were transcriptionally profiled. Constructs A and C were effective at knockdown of CLCA4 while B and a nonsilencing control were not (Figure 5A). In addition, the knockdown cell lines A and C also displayed dramatic downregulation of the epithelial marker E-cadherin and upregulation of mesenchymal markers vimentin and fibronectin relative to nonsilencing controls and parental HMLE. This apparent EMT was confirmed at the protein level by immunoblot, except that fibronectin was not upregulated in H4C (Figure 5B). We have previously observed variability in fibronectin upregulation in response to knockdown of CLCA2 [16]. This may represent a difference in completeness of EMT with different knockdown constructs or differences in fibronectin solubility due to polymerization [23].

We showed previously that induction of EMT in HMEC by several methods invariably resulted in downregulation of CLCA2. Accordingly, we found here that knockdown of CLCA4 also downregulated CLCA2, suggesting cooperative action of the two genes in epithelial differentiation (Figure 5C).

**Figure 6:**
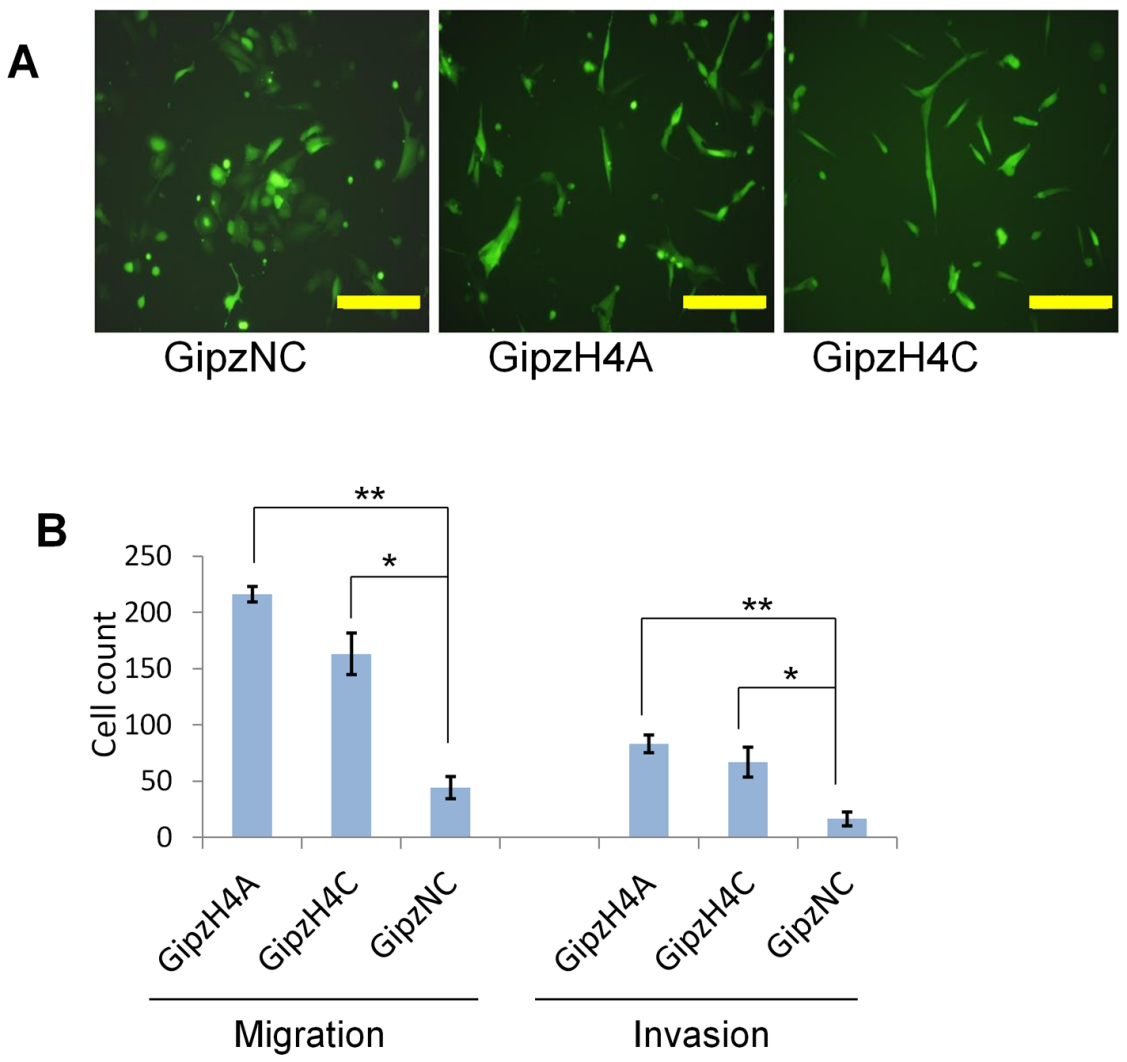
Knockdown of CLCA4 promotes cell migration and invasion. **A**, microimages showing loss of cobblestone and acquisition of hummingbird morphology with knockdown. Bar, 200 microns. **B**, Boyden chemotaxis chamber migration assay (left) and invasion assay (right).

The shift to a mesenchymal program was reflected in cell morphology and behavior. Knockdown cell lines A and C became more spindle-like and lost cobblestone morphology, while controls did not (Figure 6A). The cells also became more migratory and invasive in chemotaxis chambers (Figure 6B). These results suggest that CLCA4 plays an essential role in maintenance of epithelial differentiation.

**Figure 7:**
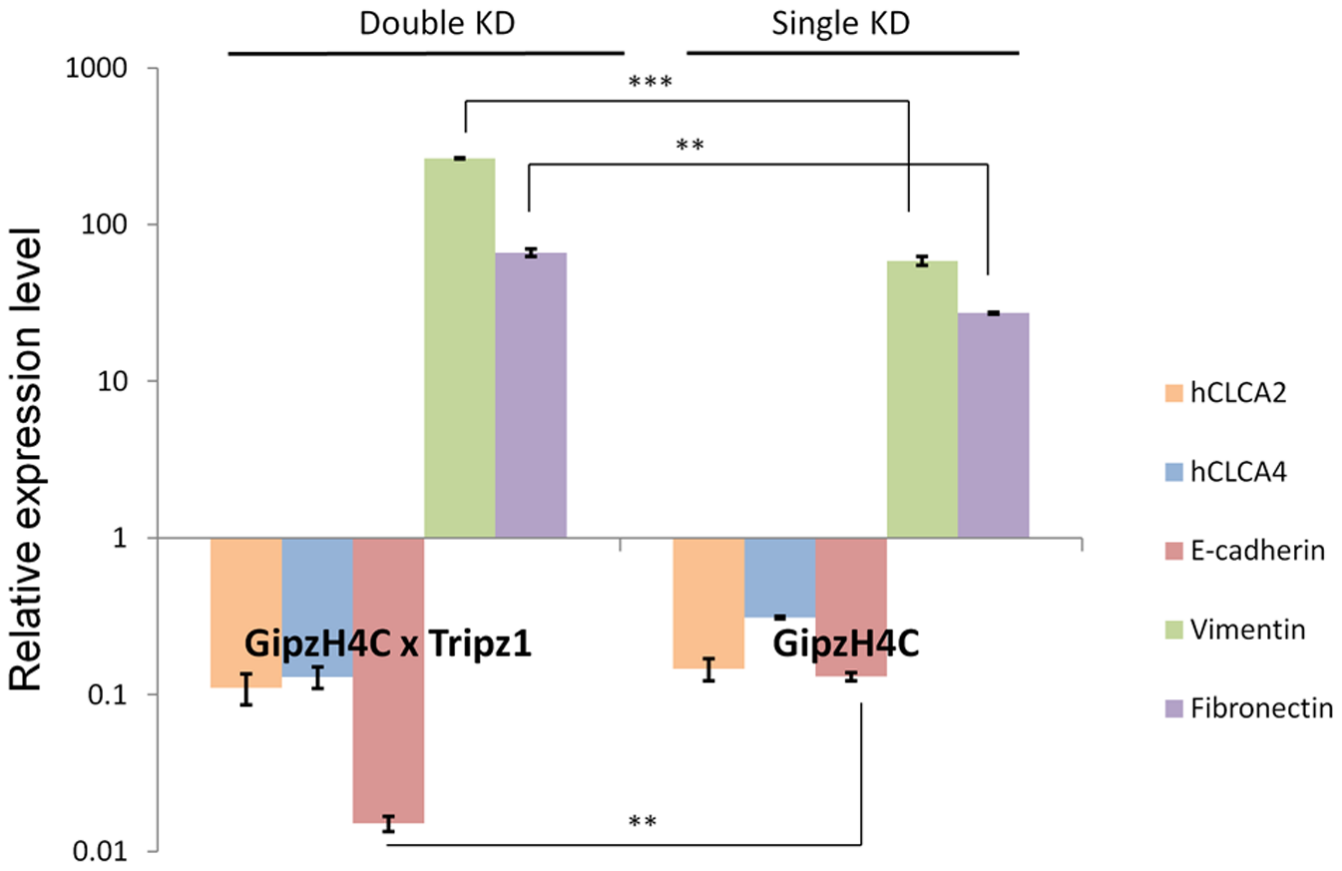
Double knockdown of CLCA4 and CLCA2 enhances EMT. HMLE cell lines bearing CLCA4 knockdown constructs were transduced with the CLCA2 knockdown construct Tripz1. Cells were selected in the presence of puromycin and doxycycline for two weeks. At least 90% of Tripz1-infected cells expressed RFP. Values were normalized to the non-silencing control, GipzNC.

That EMT occurs in response to knockdown of either CLCA2 or CLCA4 indicates that they play distinct, nonredundant roles in epithelial differentiation. Thus, double knockdown would be expected to enhance the EMT profile relative to single knockdown cells. We tested this prediction by transducing a CLCA2 knockdown construct, Tripz1, into the previously established CLCA4 knockdown cell line H4C [16]. Indeed, double knockdown enhanced downregulation of E-cadherin and upregulation of vimentin and fibronectin (Figure 7).

### Loss of CLCA4 predicts lower relapse-free survival of basal-like and luminal B breast cancers

**Figure 8:**
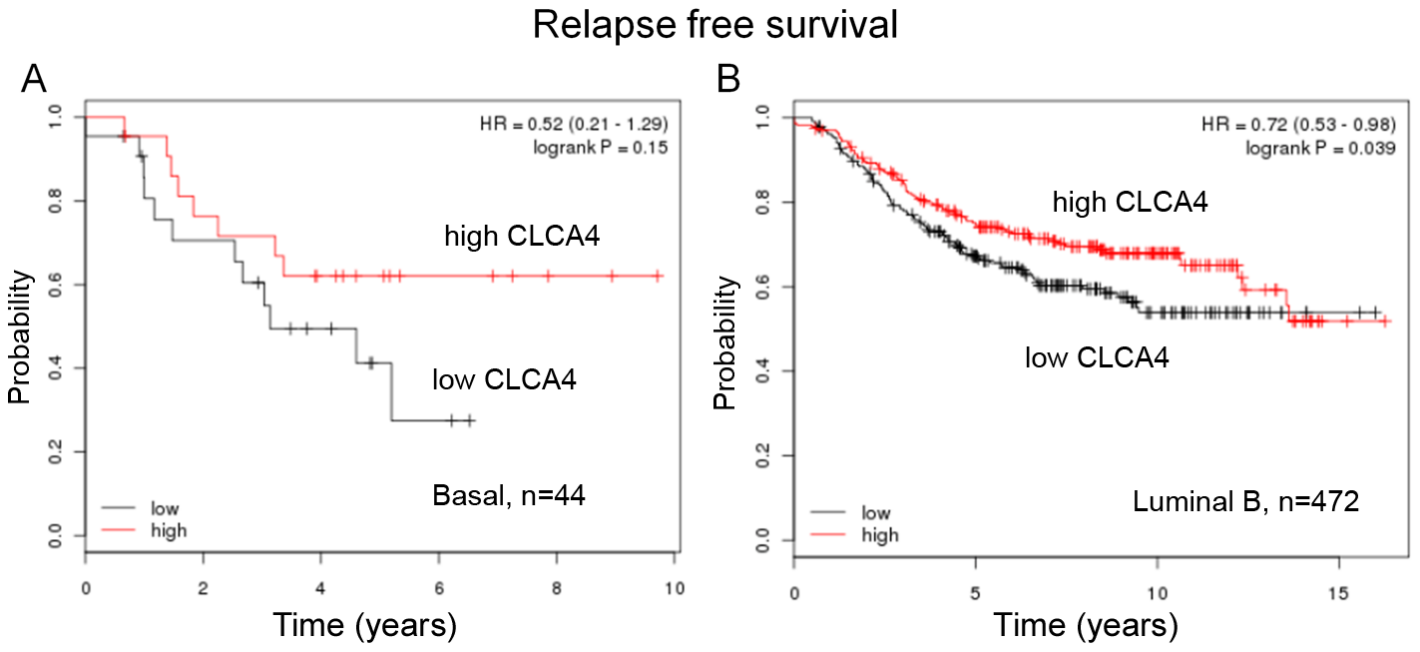
CLCA4 and relapse-free survival in breast cancer patients. Kaplan-Meier plots of relapse-free survival of patients with (A) basal-like, ER-, PR-, LN+ or (B) luminal B breast cancer relative to CLCA4 expression. Upper curve, red, indicates higher than median expression, and lower curve, black, lower than median expression.

Basal breast cancers that have lost epithelial markers are among the most likely to recur and are resistant to chemotherapy [3]. We used online resources to assess whether loss of CLCA4 signaled a poor prognosis in this subtype [24]. We found loss of CLCA4 indicated lower relapse-free survival in basal breast cancers that were negative for both estrogen receptor and progesterone receptor and lymph-node positive (Figure 8A). Because of the limited number of patients, the data did not reach statistical significance. However, CLCA4 did confer a statistically significant relapse-free survival advantage to patients with Luminal B breast cancers, a subtype that is generally low in estrogen and progesterone receptors and prone to relapse (Figure 8B) [3].

## Discussion

Maintaining epithelial differentiation requires the concerted action of a multiplicity of functionally diverse proteins, among them transcription factors, cell junctional adhesion molecules, and ion channels [8], [12], [25]–[28]. Studying the mechanisms that limit epithelial proliferation and maintain differentiation has revealed important tumor suppression mechanisms [8], [25]. In our previous work, we demonstrated that CLCA2 is a stress-inducible inhibitor of cell proliferation that plays a critical role in differentiation of mammary epithelium [15], [16]. Here we asked whether CLCA4 has a similar function. The results indicate that CLCA4 is also induced by stress and by conditions that promote epithelial differentiation such as growth on permeable membranes or suspension in extracellular matrix; that CLCA4 is required to maintain differentiation, as its attenuation caused EMT; and that ectopic expression of CLCA4 similarly inhibits proliferation of breast cancer cells, as transduction of either CLCA2 or CLCA4 into MCF7 resulted in microcolonies of enlarged cells.

The human genome encodes three functional CLCA proteins, CLCA1, CLCA2, and CLCA4 [13]. Originally thought to be chloride channels, they are currently treated as accessory proteins to a yet unidentified chloride channel [29]. Structurally, we have shown that CLCA2 is a Type I transmembrane protein that is cleaved at the cell surface near amino acid 700 to produce a 100 kDal soluble ectodomain and a membrane-anchored C-terminus. CLCA4 has a similar structure to CLCA2, while CLCA1 lacks a transmembrane segment [30]. Others have demonstrated that the N-terminus contains a metalloprotease domain that is responsible for the cleavage event [29], [31], [32]. The ectodomain is proposed to bind and activate the unknown channel [29].

CLCA4 and CLCA1 are both expressed at highest levels in colon, and both are dramatically downregulated in colon cancer [17], [18]. Recently, CLCA1 was found to play a major role in differentiation of colonic epithelial cells [18]. In these studies, differentiation could be induced by sodium butyrate treatment or allowing cells to grow beyond confluency in two-dimensional culture. CLCA1 expression was induced by both conditions, while knockdown of CLCA1 prevented differentiation. These results parallel ours with CLCA2 and CLCA4 in breast. It will be interesting to determine whether double knockdown in colon will exacerbate the loss of epithelial differentiation as we observed in mammary epithelial cells.

Recently, different chloride channels have been shown to have either tumor-promoting or tumor-suppressive roles [27], [28], [33]. For example, the anoctamin 1 (ANO1) gene encodes a calcium-activated chloride channel that is frequently amplified or upregulated in breast, prostate, and head-and-neck cancers [33]–[35]. Breast cancer cells overexpressing it had a proliferative advantage, while knockdown or pharmacological inhibition of ANO1 in those cell lines reduced colony size in vitro [33]. ANO1 also enhanced migration in one study [35]. In breast cancer, these effects were associated with increases in signaling by the epidermal growth factor receptor (EGFR) and calmodulin-dependent protein kinase II (CamKII).

In contrast to ANO1, another chloride channel behaves as a candidate tumor suppressor in breast cancer. The Cystic Fibrosis Transmembrane Conductance Regulator, CFTR, is a cAMP-responsive channel for chloride and bicarbonate; mutations in CFTR are responsible for cystic fibrosis [36]. CFTR is also frequently downregulated in breast and prostate cancers [27], [28]. Moreover, knockdown of CFTR expression or pharmacological inhibition of its channel function is sufficient to induce EMT in a breast cell line, indicating that channel function is necessary for epithelial differentiation [27]. Intriguingly, sequence polymorphisms in the CLCA4 promoter have recently been found to modify the severity of cystic fibrosis disease [37]. It is tempting to speculate that CLCA4 promotes epithelial differentiation by modulating CFTR conductance or downstream signaling. In addition or alternatively, CLCA proteins may have other targets in cellular membranes that could explain their effects on cell proliferation and differentiation. Future studies will address these possibilities.

## Materials and Methods

### Ethics Statement

The research described herein meets all applicable standards for the ethics of experimentation and research integrity. Human cancer data were derived from public databanks or cell lines that are exempt from IRB requirements. The cell lines HMLE, HMLEN and HMLER were kind gifts from Robert Weinberg (MIT). Their construction from anonymous, commercially available human mammary epithelial cells (Clonetics) has been published [38].

### Cell lines and cell culture

HEK-293T, breast cancer cell lines BT-549, MCF7, MDA-MB-231, and colon cancer cell line HCT116 were obtained from ATCC and cultured as directed. HMLE, HMLEN and HMLER were kind gifts from Robert Weinberg (MIT). Their construction from anonymous, commercially available human mammary epithelial cells (Clonetics) has been published [38]. HMLE was immortalized by transduction of hTERT and the early region of SV40; HMLEN was transduced with Her2 in addition; HMLER was transduced with activated K-rasV12 [38].

MCF10CA1d (referred to herein as CA1d) were obtained from the Barbara Ann Karmanos Cancer Institute and were cultured in DMEM plus 10% FBS. Knockdown of CLCA4 was obtained by GIPZ lentiviral transduction of shRNA (GipzH4A (24656), GipzH4B (24660) and GipzH4C (24661)) from Open Biosystems. Transduced cells were selected with puromycin (1 µg/ml) for two weeks before extracting RNA and protein.

### Bioinformatics

Oncomine (Compendia Bioscience, Ann Arbor, MI), Gene Expression Omnibus (NCBI) and Nextbio were used to analyze CLCA4 gene expression patterns in breast cancer and colorectal cancer. The patient survival data were obtained from Kaplan-Meier Plotter [24].

### RNA extraction and RT-qPCR

Cells were grown to confluency and harvested using Trizol (Invitrogen). RNA was extracted and reverse transcribed as described [15]. Expression was quantified by qPCR using an ABI7500 instrument. Primer sequences are available upon request.

### Plasmid construction and colony formation assay

CLCA4 cDNA was obtained from Open Biosystems. A Flag tag was inserted into CLCA4 sequence at nucleotide position1782 after the start codon. The CLCA4-flag was then transferred to pLEX lentivirus and packaged as described [16]. 5,000 cells (MCF7, CA1d and HCT116) were seeded into a 6 well plate and infected. The pLEX empty vector was also packaged and used as a positive control. 72 h after infection, cells were selected using puromycin at 1 µg/ml for 7 days (CA1d and HCT116) or 14 days (MCF7). Then cells were fixed and stained using crystal violet as described [16].

### Western blot analysis

For western blots, whole cell lysates were prepared from confluent cells, protein concentration was measured using the BCA assay, and 50 µg of protein was loaded per lane. CLCA4-Flag was detected using M2 anti-Flag antibody (Agilent). Antibodies for E-cadherin and fibronectin were from Cell Signaling, vimentin from Millipore, and actin from PhosphoSolutions. The size marker was Dual color (Bio-Rad). Protein expression was quantified on an Odyssey instrument (Licor).

### Differential trypsinization

HMLE cells were grown and differentially trypsinized as described [22], followed by RNA extraction and analysis as described above.

### Migration and invasion assays

Migration was measured by seeding 10^5^ cells onto Boyden chamber inserts with 8 um pore size in a 24 well plate. The insert contained cells in serum-free medium while the well contained medium with 5% FBS. After 6 h, the inserts were stained, and cells present on the lower side of the membrane were quantified by counting four different fields at 150× magnification. The invasion assay was done in a similar way using the modified Boyden chamber containing Matrigel coated membranes (BD Biosciences).

### Statistics

A minimum of three replicates were analyzed for each experiment presented. Data are presented as the mean ±s.e.m. Student's t test was used to assess the statistical significance. *P<0.05; **P<0.01 and ***P<0.001.

## Supporting Information

Figure S1 **Clonogenicity assays in breast cancer cell line CA1d and colon cancer cell line HCT116.** CLCA4 and pLex vector were packaged and transduced into cells, and colonies were selected with puromycin for 7 days then stained with crystal violet in methanol.
(TIF)
