# Supplementary figures and images for: Loss of CLCA4 Promotes Epithelial-to-Mesenchymal Transition in Breast Cancer Cells
Source: PLoS One. 2013 Dec 26;8(12):e83943. doi: 10.1371/journal.pone.0083943 (PMC3873418; doi:10.1371/journal.pone.0083943)

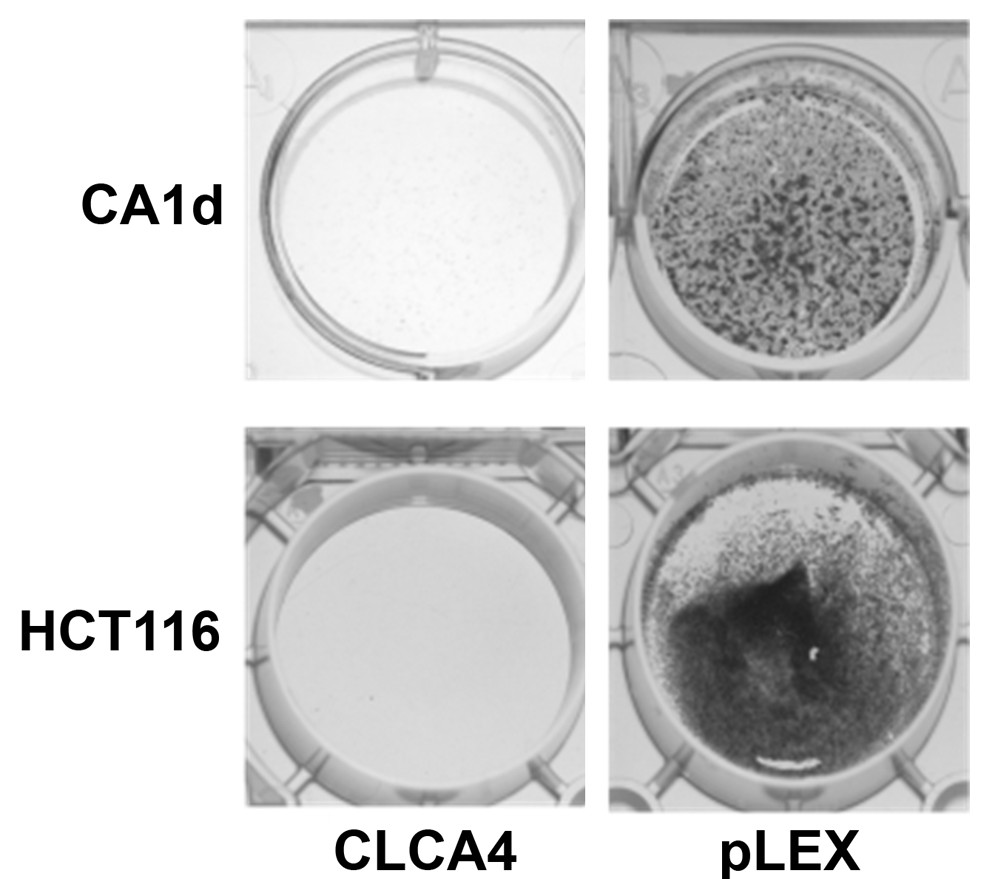

Supplement: Figure S1 — Clonogenicity assays in breast cancer cell line CA1d and colon cancer cell line HCT116. CLCA4 and pLex vector were packaged and transduced into cells, and colonies were selected with puromycin for 7 days then stained with crystal violet in methanol. (TIF) [file pone.0083943.s001.tif]
